# Fluff-thieving birds sabotage seed dispersal

**DOI:** 10.1098/rsos.160538

**Published:** 2017-01-11

**Authors:** Vanya G. Rohwer, Anton Pauw, Paul R. Martin

**Affiliations:** 1 Department of Biology, Queen's University, Kingston, Ontario, CanadaK7 L 3N6; 2 Department of Botany and Zoology, Stellenbosch University, Private Bag X1, Matieland 7602, South Africa

**Keywords:** *Eriocephalus*, Karoo prinia, mutualism, parasite, seed dispersal

## Abstract

Characterizing many species interactions as mutualisms can be misleading because some members of the interaction derive greater fitness benefits at the expense of other members. We provide detailed natural history data on a suspected bird–plant mutualism in South Africa where many species of birds use fluffy *Eriocephalus* seed material to construct their nests, potentially dispersing seeds for the plant. We focus on a common bird, *Prinia maculosa*, which invests heavily in gathering *Eriocephalus* material. Prinias spent 5 of their median 6-day nest construction period adding seed material to their nests and frequently travelled outside their territory boundary to gather *Eriocephalus* material. Yet, prinias gathered primarily *Eriocephalus* fluff and actively avoided gathering seeds. The average prinia nest contained only 6.6 seeds, but contained fluff from 579 seeds. These data suggest that prinias provide limited dispersal benefits to *Eriocephalus* plants. By contrast, the large amounts of *Eriocephalus* fluff in prinia nests, and the effort that prinias invest in gathering it, suggest that prinias benefit from constructing their nests with *Eriocephalus* material. We end by outlining hypotheses for possible fitness benefits that *Eriocephalus* material could provide prinias and other birds.

## 1. Introduction

Mutualisms—interspecific interactions that benefit both species—play an important role in ecological communities and in shaping the evolutionary trajectories of interacting species [1,2]. Although many interactions are classically viewed as mutualisms, detailed study of these interactions often reveals non-reciprocal, or at best, highly asymmetrical interactions where one party derives greater fitness benefits from the interaction than the other [3–6]. Fitness outcomes of mutualisms can vary across space and time, depending on trait values of local species [7] and the community context where the interaction occurs [8]. Changes in the community composition can alter the nature of mutualistic interactions, changing selective pressures on interacting species [9] or leading to the breakdown of mutualisms [10]. Mutualisms are further complicated by the number of species involved in the interaction [11]. Mutualists typically form multispecies guilds that almost always include poor quality mutualists or cheaters that reap rewards without providing goods or services in return [12–14]. All these cases highlight how detailed study of species interactions typically viewed as mutualisms can lead to a more complex and dynamic understanding of species interactions and their fitness outcomes. Despite the importance of mutualistic interactions in ecological communities [15,16], we know little about most interactions.

**Figure 1.**
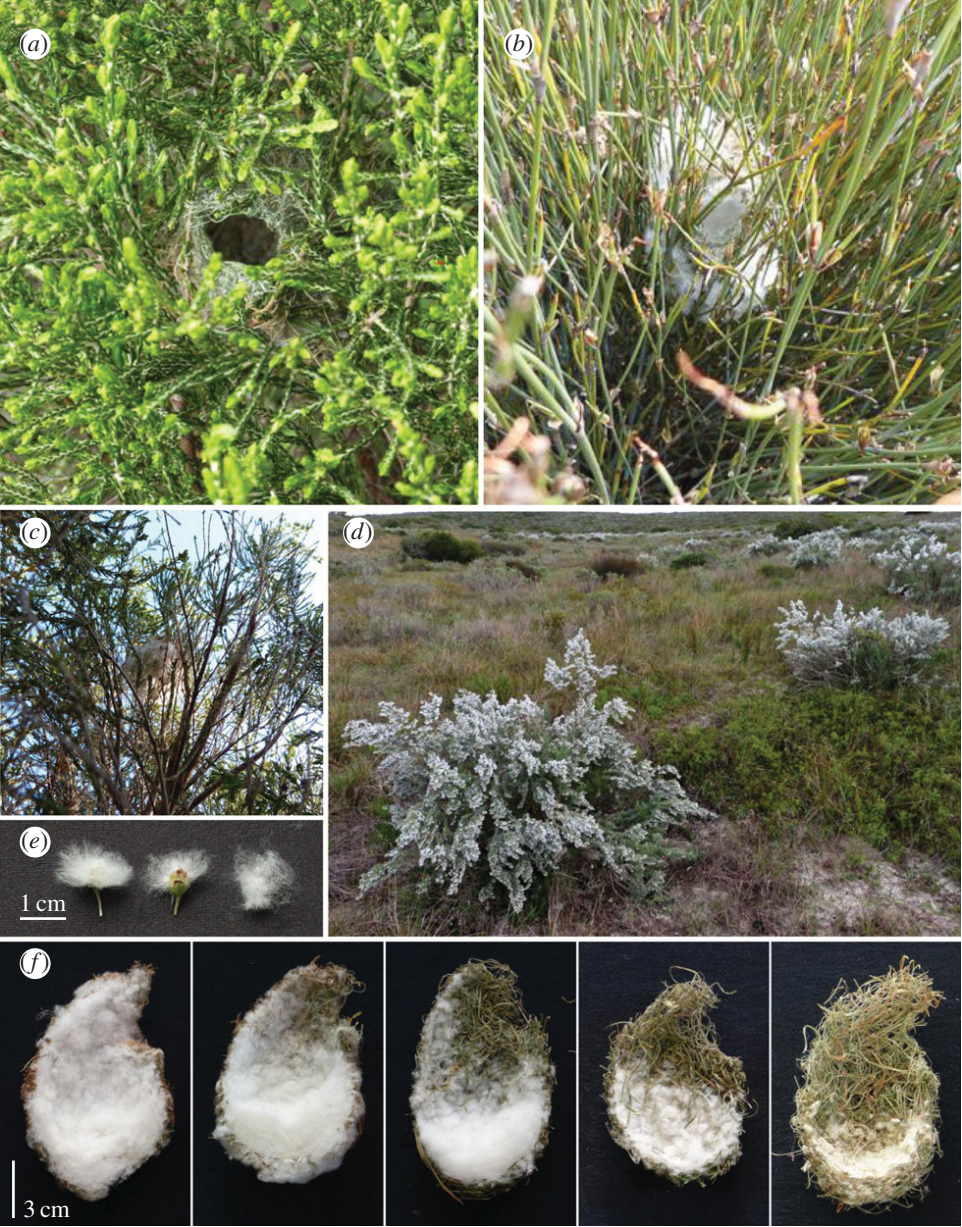
*Eriocephalus* material in Karoo prinia nests. Karoo prinia nests constructed without (*a*) and with an abundance (*b*) of *Eriocephalus* material. *Eriocephalus* material may help conceal nests—photo (*c*) is taken from the ground looking up at the base of a Karoo prinia nest (nest is in centre of the photo). (*d*) *Eriocephalus* bush. (*e*) *Eriocephalus* seed head with fluff (left), half of fluff missing (plucked by authors) (middle), and fluff removed from seed head (right). (*f*) Five Karoo prinia nests cut in half (nest entrance is in the upper right in each photo), with a range of *Eriocephalus* material on the nest interior. The left four nests all have *Eriocephalus* material; the right most nest has none and is lined primarily with *Trichocephalus stipularis* fluff and *Helichrysum* spp. leaves (all photographs: V.G.R.).

Here, we examine a suspected mutualism between birds and plants in southern Africa. In this system, many species of birds use seed material from plants in the genus *Eriocephalus* to construct their nests [17]. *Eriocephalus* plants are highly aromatic [18] and produce many small seed heads that are surrounded by white, cotton-like fluff (figure 1). Birds that incorporate *Eriocephalus* seed material into their nests may benefit in diverse ways. For example, the chemical compounds in *Eriocephalus* seed material could reduce nest ectoparasites or nest predation from predators that rely on olfactory cues to locate nests. Similarly, the pale fluff surrounding *Eriocephalus* seed material could improve nest microclimate or help conceal nests from visual predators, increasing the reproductive success of birds. *Eriocephalus* plants may also benefit from interactions with birds. Plants that have their seed material incorporated into the nests of birds may benefit by enhanced seed dispersal and increased seed survival and germination [17,19].

We provide detailed natural history data on the interactions between *Eriocephalus* plants and birds in Western Cape Province, South Africa, with a focus on phenology, bird behaviour and seed dispersal. We focus on the most abundant species of *Eriocephalus* at our site—*E. racemosus*—and the most abundant bird species breeding at our site—the Karoo prinia (*Prinia maculosa*). In addition to their abundance, Karoo prinias use large amounts of *Eriocephalus* seed materials to construct their nests, and are thus expected to be one of the most important bird species interacting with *E. racemosus* at our site. We first describe the breeding phenologies of Karoo prinia relative to the phenology of seed material production of *E. racemosus*. If interactions between prinias and *Eriocephalus* are mutually important, then the phenologies of bird nest building and plant seed production should correspond with each other. Second, we document the time spent and distance travelled by prinias to gather *Eriocephalus* material for their nests. If prinias invest considerable time and effort gathering *Eriocephalus* material, then using this material probably confers some benefit to prinias. To further understand how *Eriocephalus* material may benefit prinias, we also describe where in the nest prinias place the bulk of *Eriocephalus* material. Third, we quantify the number of *Eriocephalus* seeds in Karoo prinia nests to assess whether prinias are effective agents of dispersal for *Eriocephalus* seeds. If nest building prinias are important for *Eriocephalus* seed dispersal, then their nests should contain many seeds.

## 2. Material and methods

### 2.1. Study site

The Koeberg Nature Reserve (33°41′ S, 18°27′ E) is approximately 35 km north of Cape Town, along the west coast of South Africa. Koeberg has a Mediterranean climate with warm dry summers and cool wet winters (electronic supplementary material, figure S1). Temperatures at Koeberg are moderated by the Atlantic Ocean. Winter temperatures range from 10°C to 20°C, while in summer, cold waters of the Bengula current keep temperatures between 15°C and 25°C [20]. Winds are strong (year-round average wind speed 3–4 m s^−1^), persistent, and typically from the south, further cooling temperatures during the summer [20]. Two dominant types of vegetation occur at Koeberg: (i) dune thicket vegetation, a dense often impenetrable tangle of shrubs and (ii) sand plain fynbos vegetation, which tends to be more open with isolated shrubs. Both vegetation types are generally short (less than 2 m), and in addition to *E. racemosus*, dominant plants include: *Passerina vulgaris*, *Chrysanthemoides incana*, *Rhus leavigata*, *Euclea racemosa*, *Trichocephalus stipularis*, *Muraltia spinosa*, and multiple species of Restionaceae [21]. Koeberg supports a diversity of small passerine birds, many of which use *Eriocephalus* material to construct their nests [22]; in addition to Karoo prinias, common breeding birds include: grey-backed cisticola *Cisticola subruficapilla*, southern double-collared sunbird *Cinnyris chalybeus*, chestnut-vented tit-babbler *Sylvia subcaeruleum*, Cape white-eye *Zosterops capensis*, Cape bulbul *Pycnonotus capensis*, Cape weavers *Ploceus capensis*, white-backed mousebird *Colius colius*, yellow canary *Crithagra flaviventris* and common waxbill *Estrilda astrild*. Most of these species nest in low shrubs, share the challenges of maintaining consistent nest temperatures, and have similar nest predators. Nest predators at Koeberg include a diversity of birds (pied crow *Corvus albus*, fiscal shrike *Lanius collaris*, bokmakierie *Telophorus zeylonus*), mammals (Cape grey mongoose *Galerella pulverulenta,* Southern African vlei rat *Otomys irroratus*, four-striped grass mouse *Rhabdomys pumilio*) and snakes (rhombic egg-eater *Dasypeltis scabra*, boomslang *Dispholidus typus*, Cape cobra *Naja nivea*). Field observations of depredated nests suggest that rhombic egg-eating snakes and pied crows are the dominant nest predators of small passerines [23–26] (V.G.R. 2013, personal observations). Brood parasites (e.g. Klaas's cuckoo *Chrysococcyx klaas*, pin-tailed whydah *Vidua macroura*) are present but uncommon, and we found no brood parasite eggs in over 200 nests of common small passerines at Koeberg. All nests were found in an area roughly 3 × 3 km in the Koeberg Nature Reserve.

### 2.2. Karoo prinia

Karoo prinias are small (approx. 10 g), non-migratory passerines (Passeriformes: Cisticolidae) native to southern Africa and typically have high adult survival and low nesting success [27,28]. They are sexually monochromatic, have long-term pair bonds, and maintain year-round territories (average territory diameter on our sites approx. 85 m) that are aggressively defended during the breeding season [29].

Karoo prinias breeding at Koeberg often place their nests low (less than 1 m) [24] in *Passerina* shrubs and Restionaceae. Females are the primary builders of the nest's grass frame, and both males and females help line the nest with downy plant material, often bringing *Eriocephalus* fluff when it is available [29] (V.G.R. 2013, personal observations). Females lay one egg per day, but may delay egg deposition during periods of cold weather. Only females incubate and males make few feeding visits to incubating females [30]. Both males and females brood newly hatched young (usually until nestling day 7), feed young, and provide post-fledging care for two to three weeks after young leave the nest [29]. Pairs are socially monogamous. Extra-pair copulations are probably rare because the seminal vesicles of Karoo prinias are small (V.G.R. 2013, personal observations), like those found in species with low extra-pair paternity and sperm competition [31].

At Koeberg, nest predation is the primary cause of nest failure and Karoo prinias face some of the highest daily nest predation rates recorded for passerine birds (7.5% daily nest predation [24,32]).

### 2.3. *Eriocephalus* plants

*Eriocephalus* (Asteraceae; wild rosemary) are highly aromatic, perennial shrubs native to southern Africa [18]. In the most recent taxonomic treatment of this group, Müller *et al*. [33] recognized 32 species within the genus *Eriocephalus* based on morphology. Two species of *Eriocephalus* occur at our study site: *E. racemosus* and *E. africanus*. *E. racemosus* is far more common than *E. africanus* and our analyses of *Eriocephalus* phenology, material use in prinia nests, and prinia–*Eriocephalus* interactions refer to *E. racemosus* exclusively. *E. racemosus* is common in open sand plain habitats at low elevations (less than 100 m.a.s.l.) primarily along the southern coast of South Africa from Lambert's Bay to Port Elisabeth [33]. Plants reach approximately 2 m in height at maturity and often have irregular growth patterns and flimsy branches.

Flowering occurs in spring (August–September at Koeberg). Typical of Asteraceae, several flowers are grouped together into a capitulum surrounded by involucral bracts. The capitulum (seed head) is approximately 2 mm in diameter and contains approximately 3 white ray florets and approximately 7 purple disc florets. Unusual for Asteraceae, the seeds are retained inside the capitulum, which is dispersed as a single unit. After flowering, very long silky trichomes grow from the involucral bracts surrounding the capitulum (figure 1). The pappus, which normally aids wind dispersal in the Asteraceae (e.g. dandelions), plays no role in seed dispersal. Mature *E. racemosus* plants produce hundreds to thousands of seed heads, giving plants an overall pale appearance (figure 1).

For our study, we distinguish two parts of *Eriocephalus* seed material: the ‘seed head’ and the fluffy exterior surrounding the seed head (henceforth ‘fluff’) (figure 1). While a single seed head contains several individual seeds (like many plants in the Asteraceae) [33], we counted seed heads only because this is the part of the plant with which birds interact.

### 2.4. Reproductive phenologies of Karoo prinias and *Eriocephalus*

To compare the reproductive phenologies of Karoo prinias and *Eriocephalus*, we monitored breeding activity of prinias and the development of *Eriocephalus* seed material during the austral spring (August–November) of 2013.

#### 2.4.1. Breeding phenology of Karoo prinias

We found Karoo prinia nests by watching adults carry material to their nests and by searching suitable habitat. Upon finding a nest, we marked its location with a handheld GPS receiver (GPSmap 60, Garmin International Inc., Olathe, KS, USA) and checked it every 3 days [34–36]. We recorded or estimated first egg date for all nests using the following methods: nests found during the building stage were monitored until the first egg was laid; for nests found during the laying period, we back-counted one egg per day to the first egg (Karoo prinias typically lay one egg per day until the clutch is complete and begin incubation on the day that the last egg is laid [29]); for nests found during incubation, we either back-counted from the hatch date (by 14 days for incubation plus the appropriate number of days, based on clutch size, assuming that females laid one egg per day [29]) or, for clutches that did not hatch, we took the midpoint between the least and most advanced possible stage of incubation [36].

#### 2.4.2. Phenology of *Eriocephalus*

We surveyed three sites with *Eriocephalus* plants in the Koeberg Nature Reserve to monitor the development of fluff; sites were separated by 500–1500 m, and each site contained approximately 500 plants. We scored phenology for over 100 plants at each site using eight categories that ranged from least to most developed: bud, early flower, peak flower, late flower, sparse fluff, early fluff, medium fluff and thick fluff (see electronic supplementary material, table S1, for descriptions of categories). We examined all of the flowers/seed heads on each plant and then categorized the plant into the phenological category that characterized more than 50% of its flowers/seed heads. We visited sites once every 10 ± 1 days for 86 days (starting 24 August 2013, ending 18 November 2013), and stopped when all plants in a patch had thick fluff and had lost roughly half of their seed material.

#### 2.4.3. Ease of seed head removal from *Eriocephalus*

As another measure of *Eriocephalus* phenology, we estimated the ease by which seed heads could be removed from plants as a function of season by pulling on the fluff of a seed head with forceps at three different dates (26 August; 1 October; 3 November), corresponding to early, middle and late breeding times for Karoo prinias in 2013. We simulated fluff-picking behaviour of prinias using forceps that had a surface area similar to the beaks of prinia and other small passerine birds. During each seed head removal trial, we plucked at the fluff of 100 *Eriocephalus* seed heads from 10 different plants (1000 seed heads in total for each plucking date) and counted the number of plucks that resulted in the removal of a seed head from the plant. We marked all plants with aluminium tags and returned to each plant for all subsequent picking trials. We targeted seed heads from the entirety of each plant (rather than select branches), in case seeds on some branches matured faster than others.

We tested for differences in the number of seed heads removed from plants between each seed-removal trial using a linear mixed effects model in R [37]. The number of seed heads removed was our dependent variable, date of each seed-removal trial was our predictor variable, and individual plant identification was a grouping variable to account for multiple measures originating from the same plant. Prior to analysis, we transformed the number of seed heads removed using [natural logarithm(number of seeds removed + 1)], because the distribution of these data was right skewed; this transformation helped normalize the distribution so that the data better fit the assumptions of our model. We checked that model residuals did not deviate from normality using a Shapiro–Wilk test, and that residual model variance did not differ significantly between time intervals using a Bartlett's test, following Zuur *et al*. [38].

### 2.5. Karoo prinia distance travelled and time spent gathering *Eriocephalus* material

We measured the distance that prinias travelled to gather *Eriocephalus* material by watching focal birds gather material and then return to their nests. For each observation, we made two measurements (in metres) using a GPS receiver: (i) the straight-line distance from the nest to the plant from which prinias gathered material and (ii) the straight-line distance from the nest to the closest *Eriocephalus* plant that was at a similar phenological stage as the plant from which prinias gathered material. We then compared these two distances to test if prinias gathered material from *Eriocephalus* plants closest to their nests.

For all nests (including those that we did not observe during the building period), we measured the proximity (in metres) to the closest *Eriocephalus* bush to estimate the minimum distance required to gather *Eriocephalus* material.

We assessed the time spent gathering *Eriocephalus* material in two ways. First, we watched focal birds and measured the time (in seconds) spent perched in *Eriocephalus* plants while gathering a single load of material. We used a stopwatch and defined the time spent gathering material as the interval between the first and last picks at *Eriocephalus* fluff during a single visit to an *Eriocephalus* plant, which roughly corresponded to when the bird arrived and departed from the plant. Our second method estimated the time (in days) allocated to nest construction and lining the nest with *Eriocephalus* material, as the number of days between the start of nest construction (the appearance of the first green strands of grass) until the first egg date. We measured the time allocated to lining the nest with *Eriocephalus* fluff as the number of days between the completion of the grass frame and the appearance of the first egg. Although some Karoo prinias begin lining the nest prior to completion of the grass frame (V.G.R. 2013, personal observations), this time interval encompasses the majority of the nest-lining process.

### 2.6. Distribution and amount of *Eriocephalus* fluff in prinia nests

We quantified the distribution of *Eriocephalus* material in Karoo prinia nests by cutting nests in half and measuring nest-wall thickness at 10 evenly spaced locations around the nest (see electronic supplementary material, figure S3). We scaled these 10 measurements to landmarks on each nest (e.g. the nest entrance, base of the nest and roof of the nest), making nest-wall measures from different nests comparable. We measured wall thickness using a single half of the nest, and summarized these data using boxplots.

We quantified the amount of *Eriocephalus* fluff in four ways: (i) measuring the depth of fluff in the base of nests that were cut in half, (ii) photographing the interiors of all cut-in-half nests and quantifying the proportion of interior nest area covered in pale-coloured materials (mostly, but not exclusively, *Eriocephalus* fluff) using ImageJ [39], (iii) examining the amount of *Eriocephalus* material on the exterior of the nests, and (iv) weighing the total amount of *Eriocephalus* material in each nest (see the electronic supplementary material for more details).

### 2.7. *Eriocephalus* seed heads in Karoo prinia nests

To assess the effectiveness of Karoo prinias as seed dispersers, we counted the number of *Eriocephalus* seed heads and weighed the total amount of *Eriocephalus* fluff in each nest. All measurements were made from nests collected once they were inactive (i.e. monitored nests that were recently depredated, abandoned or fledged young). The condition of inactive nests was variable—some were completely destroyed with material scattered throughout the nest bush, while others were perfectly intact. Although destroyed nests could help disperse seeds, we excluded these nests from this analysis because we could not be certain that the number of seed heads in them after destruction was representative of the number of seed heads originally in the nest.

Many other bird species breeding at Koeberg use *Eriocephalus* seeds and fluff in nest construction, and could also disperse *Eriocephalus* seeds. Thus, we compared the number of *Eriocephalus* seed heads found in Karoo prinia nests with 20 nests from seven other species at our site: southern double-collared sunbird (*n *= 3), grey-backed cisticola (*n *= 9), chestnut-vented tit-babbler (*n *= 1), Cape white-eye (*n *= 1), Cape bulbul (*n *= 3), yellow canary (*n *= 2) and bokmakierie (*n *= 1). We tested for differences in the number of *Eriocephalus* seed heads between nests of Karoo prinias and all seven other species combined using a Wilcoxon rank sum test.

### 2.8. Factors influencing the amount of seeds and fluff in Karoo prinia nests

Several factors could influence the amount of *Eriocephalus* material in prinia nests. Environmental conditions during the breeding season, bush species in which nests are placed, or nest height above the ground could be associated with *Eriocephalus* use, especially if *Eriocephalus* material buffers against environmental challenges or reduces nest failure from predators or parasites attracted to nests placed in certain locations. Timing of breeding could also influence the amount of material used in nest construction if late nesting birds have less time to gather *Eriocephalus* material compared with early nesting birds, or if the ease by which birds can gather *Eriocephalus* material changes during the breeding season. Additionally, proximity to *Eriocephalus* plants may influence the amount of material birds add to their nests. We measured six factors that could influence the amount of *Eriocephalus* seed heads and fluff in prinia nests: distance to the closest *Eriocephalus* plant, number of days the nest remained active (i.e. interval between first egg date and the date the nest was depredated, fledged or abandoned, as prinias continue to add material to their nest through the incubation and nestling periods), first egg date, minimum ambient temperature on first egg date (temperature data from a weather station at Koeberg Nature Reserve), nest height and bush species in which nests were placed. All these variables have the potential to influence the amount of *Eriocephalus* material in prinia nests and may help to elucidate any function of *Eriocephalus* material in bird nests.

We measured the number of *Eriocephalus* seed heads and the amount of fluff in Karoo prinia nests separately, using five different statistical models. We corrected for multiple comparisons in the four analyses of *Eriocephalus* fluff following Pike [40]. For three analyses (number of seeds in nests, depth of fluff and mass of fluff), the distributions of our response variable showed two peaks, one at zero and one at an integer value greater than zero. Because of these bimodal distributions, we used hurdle models with a zero-adjusted Poisson distribution in R with the package pscl [41], following Zuur *et al*. [38]. Hurdle models separately test the effects of predictor variables on the zero versus non-zero (bivariate) component of the response variable, and on variation in the non-zero component of the response variable, thus fitting the bimodal distributions of these variables [38]. For analyses of *Eriocephalus* fluff on the interior and exterior of nests, data were more normally distributed, so we used generalized linear models. For all analyses, we checked the assumptions and fit of models following Zuur *et al*. [38]. We selected the best fit models based on Akaike's information criterion corrected for small samples sizes (AICc), using the *dredge* function in package MuMIn [42]. See the electronic supplementary material for detailed statistical procedures and data transformations.

## 3. Results

### 3.1. Reproductive phenologies of Karoo prinias and *Eriocephalus*

**Figure 2.**
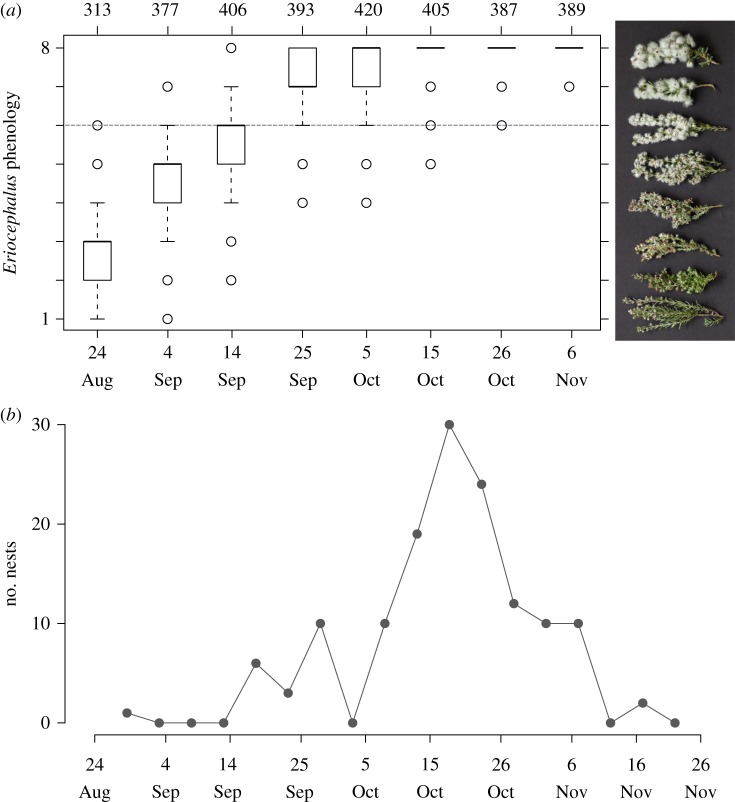
Development of *Eriocephalus* seed material and the prinia breeding season. (*a*) Progression of *Eriocephalus* seed material for three patches of plants at the Koeberg Nature Reserve; *y*-axis ranks plant development from least (1) to most (8) developed (see electronic supplementary material, table S1, for descriptions of phenological categories). Grey dotted line represents the earliest phenological stage of plants from which we observed prinias gathering fluff. Numbers above boxplots represent the number of plants surveyed, and boxplots show medians (thick lines), 25th and 75th percentiles (boxes), 1.5 times the interquartile range (whiskers), and outliers (points outside 1.5 times the interquartile range). (*b*) Time series plot of Karoo prinia first egg dates using 139 nests found during the 2013 breeding season at the Koeberg Nature Reserve.

The phenology of *E. racemosus* seed fluff production overlapped with the breeding season of Karoo prinias at the Koeberg Nature Reserve (figure 2). Fluff of *E. racemosus* plants was sufficiently developed for prinias to start using it by 14 September 2013, which coincided with first egg dates from early nests. The peak in first egg dates for Karoo prinia was approximately 20 October 2013, corresponding to the point at which nearly all *Eriocephalus* plants that we surveyed had reached the ‘thick fluff’ stage (figure 2).

The probability of seed detachment during plucking trials was strongly influenced by date (comparing glmm models with and without date: *χ*^2^ = 53.0, *p *< 0.0001); seed heads were difficult to remove early in the season, but became easier to remove as the season progressed (glmm; intercepts for each picking date differed from each other; *t* = 8.0, *p *< 0.001; electronic supplementary material, figure S2).

### 3.2. Karoo prinia distance travelled and time spent gathering *Eriocephalus* material

**Figure 3.**
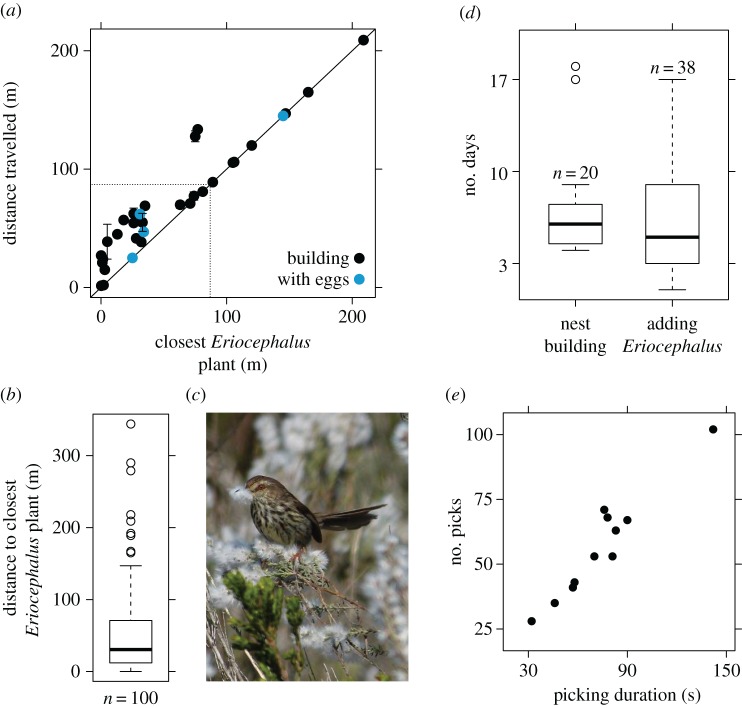
Distance travelled and time invested in gathering *Eriocephalus* material. (*a*) Summary of 83 observations from 33 different Karoo prinia nests showing the distance prinias travelled to gather *Eriocephalus* fluff regressed on the distance to the closest *Eriocephalus* plant that was in the same phenological stage as the plant from which the focal bird gathered material; multiple observations for a single nest are shown with standard deviations. Dotted lines at 83.5 m illustrate average diameter of a prinia territory. (*b*) Boxplot of the distance to the closest *Eriocephalus* bush for 100 prinia nests that contained *Eriocephalus* material. (*c*) Picture of Karoo prinia gathering *Eriocephalus* fluff from seeds held between toes. (*d*) Number of days that prinias allocated to nest building and lining the nests with *Eriocephalus* material. (*e*) The number of picks that prinias made during a single bout of material gathering regressed on the time spent perched in *Eriocephalus* bushes while gathering fluff.

Data from dissected nests (*n* = 5) and observations of prinias gathering *Eriocephalus* material then returning to their nests (*n* = 1) show that some prinias travelled over 200 m (straight-line distance between bush and nests) to gather *Eriocephalus* fluff. We watched one focal prinia travel 209 m to gather *Eriocephalus* fluff, and data from dissected nests suggest that one prinia travelled at least 344 m to gather *Eriocephalus* fluff, based on the minimum distance from their nests to the nearest *Eriocephalus* plants (figure 3; although prinias could also have salvaged material from previously constructed nests). Prinias that gathered *Eriocephalus* material from plants more than 100 m from their nests (*n *= 9) typically flew to the closest available plant, whereas birds that gathered material from plants less than 80 m from their nests (*n* = 22) did not go to the closest available plant (figure 3). These distances (80–100 m) correspond roughly to the average diameter of Karoo prinia territories (approx. 85.3 ± 18.6 m, *n* = 19; [29] and V.G.R. 2013, unpublished data), suggesting that prinias breeding on territories lacking *Eriocephalus* plants gather material from the closest available plant on neighbouring territories.

On four occasions we witnessed aggressive interactions between prinias when gathering *Eriocephalus* material; all four occasions involved individuals trespassing onto another's territory to gather fluff. On all occasions, the intruder was chased out of the defender's territory. These observations occurred during the early breeding season (before 15 September 2013) when *Eriocephalus* fluff was scarce (figure 2).

Karoo prinias spent a median of 6 days constructing their nests and 5 days lining their nests with *Eriocephalus* fluff (figure 3). *Eriocephalus* plants have flexible branches that bend easily, and prinias frequently made hovering flights to prevent falling while gathering fluff in strong winds. Prinias remained perched in the tops of moving branches for as long as two and a half minutes during a single bout of material acquisition, during which time they made as many as 102 picks at *Eriocephalus* seed heads to gather a single bill-full of fluff (see electronic supplementary material, video). When we saw prinias arrive at *Eriocephalus* bushes, they gathered fluff for an average of 62 (±34 s.d.) s, and made an average of 57 (±21 s.d.) picks (figure 3), before returning to their nest with a load of fluff. We observed prinias gathering fluff from only one bush per trip before returning directly to their nests.

### 3.3. *Eriocephalus* seed heads in Karoo prinia nests

**Figure 4.**
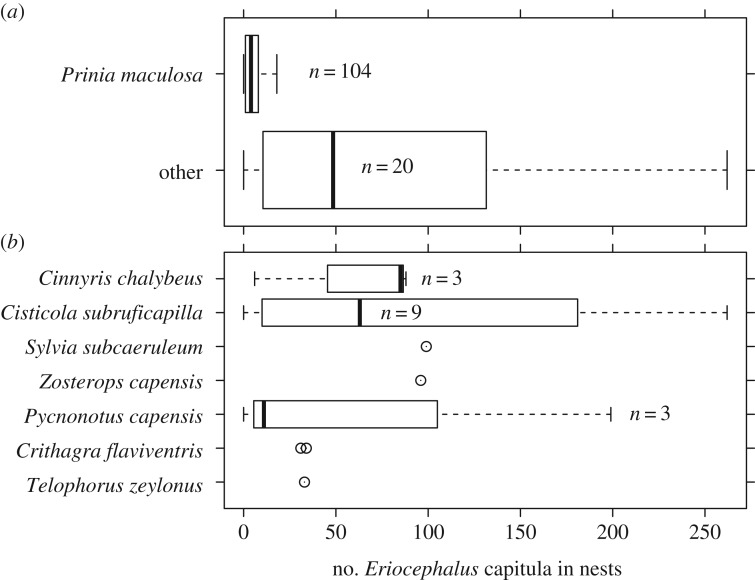
Comparison of *Eriocephalus* seed heads in bird nests from Koeberg Nature Reserve. (*a*) Nests of Karoo prinias (*Prinia maculosa*) contained fewer seeds than nests of other bird species (Wilcoxon rank sum test, *p *< 0.0001). (*b*) The number of *Eriocephalus* seed heads in the nests of the seven bird species grouped in the category ‘other’ in part (*a*) of this figure.

Prinia nests (*n* = 104) contained an average of 6.6 ± 8.0 s.d. *Eriocephalus* seed heads (range: 0–43). By contrast, the 20 nests of the other seven species contained an average of 81.0 ± 83.7 s.d. seed heads (range: 0–262; Wilcoxon rank sum test, *W* = 1768, *p* < 0.00001; figure 4).

Karoo prinias nests constructed with *Eriocephalus* fluff contained an average of 1.39 g ± 0.71 s.d. of *Eriocephalus* fluff. The average mass of fluff on the plant associated with a single seed head was 0.0024 g (±0.0019 s.d., *n* = 20); thus Karoo prinia nests contained, on average, an amount of fluff equal to that associated with 579 *Eriocephalus* seed heads.

Two behaviours suggest that Karoo prinias actively avoided gathering *Eriocephalus* seeds. First, we watched at least 10 prinias gathering fluff from seed heads that were held between their toes, which may help hold seeds in place so they are not removed with the fluff. Second, we witnessed four different prinias drop *Eriocephalus* seed heads picked while gathering fluff, instead of transporting those seeds to their nests. Dropping seed heads caused these individuals to lose their bill-full of fluff, and forced them to gather a new load of fluff before returning to their nest.

Prinias placed the majority of *Eriocephalus* fluff in the nest interior, at the base of the nest cup (electronic supplementary material, figure S3). Nest-wall thickness varied within nests and most of this variation was caused by the differential placement of *Eriocephalus* fluff; nest-wall measures taken from the top section of nests (i.e. measures 8–10 in electronic supplementary material, figure S3) were significantly thinner than those taken from the bottom section of nests (i.e. measures 3–5 in electronic supplementary material, figure S3) (Wilcoxon rank sum test, *W* = 1019.5, *p* < 0.00001, *n* = 122).

We summarize results for the factors influencing the number of *Eriocephalus* seed heads and the amount of *Eriocephalus* fluff without seeds in Karoo prinia nests in electronic supplementary material, table S2 and figure S4 (for seed heads only), and provide detailed statistical summaries of each analysis in electronic supplementary material (tables S3–S6). Overall, our analyses revealed three significant predictors of the amount of *Eriocephalus* seeds and fluff in nests: proximity of *Eriocephalus* plants, first egg date, and the number of days a nest remained active. Nests that were closer to *Eriocephalus* plants contained more fluff and seeds, later nests contained less fluff but more seeds, and nests that remained active for longer contained more *Eriocephalus* fluff in their interior, consistent with observations of Karoo prinias adding material to the nest throughout the nesting cycle [22]. Predictor variables of nest height, bush species and ambient temperature (after controlling for first egg date) were not significant in any analyses (electronic supplementary material, table S2).

## 4. Discussion

We provide some of the first detailed natural history data regarding a suspected mutualism between Karoo prinias and *Eriocephalus* plants in southern Africa. The reproductive phenologies of Karoo prinia and *E. racemosus* plants corresponded well, with seed fluff availability coinciding with nest building in prinias. Indeed, all but one of the earliest first egg dates occurred after *Eriocephalus* fluff was sufficiently developed for use in nest construction (figure 2), suggesting that *Eriocephalus* seed material is available for dispersal when birds are breeding. Karoo prinias invested considerable time and effort gathering *Eriocephalus* fluff for their nests. Prinias frequently left their territories to gather *Eriocephalus* material, travelling over 100 m and sometimes fighting with other prinias in order to access *Eriocephalus* plants. Prinias spent, on average, 1 min perched in *Eriocephalus* branches picking fluff during each material acquisition trip. The time and effort invested in gathering *Eriocephalus* material suggests that it may benefit prinias. However, closer examination of this suspected bird–plant mutualism reveals a more complex interaction, with fitness outcomes to plants probably depending on the species of birds with which it interacts. Karoo prinias actively avoided gathering *Eriocephalus* seeds by holding seed heads in place with their toes and pulling only fluff, and by dropping seed heads when they were removed from plants instead of bringing them to their nest. The fluff in the average prinia nest at our study site would have contained 579 seed heads on the plant, yet nests contained an average of only 6.6 seed heads. The few seeds in prinia nests contrasted with other species of birds that incorporated many more *Eriocephalus* seed heads into their nests (figure 4). These results suggest that Karoo prinias are poor dispersers of *Eriocephalus* seeds in general, and are particularly poor dispersers relative to some other species of birds on our site.

Why Karoo prinias avoided *Eriocephalus* seeds, while other species did not, remains unknown. Incorporating *Eriocephalus* seeds into nests might attract seed predators or parasites (e.g. ants, fungi and bacteria) that reduce prinia nesting success. Alternatively, seeds may be bulky and difficult for prinias to manipulate during nest building, or create an uneven nest lining for developing young. Gathering and transporting *Eriocephalus* seeds with the fluff could also increase the energetic costs of nest building; however, the seeds are extremely light (average dry seed head mass (g): 0.0061 ± 0.0019 s.d., *n* = 20), and prinias appeared to expend significant time and energy avoiding seeds during fluff gathering, increasing the costs of nest building.

Fitness outcomes for similar bird–*Eriocephalus* interactions probably vary across space and time. Environmental gradients vary dramatically throughout southern Africa, as do the traits of the interacting species [22,33]. In the Southern Karoo, a drier, higher elevation area several hundred kilometres east of our coastal study site, the number of *Eriocephalus* seeds in prinia nests (either *P. maculosa* or *Phragmacia substriata*) is much higher [17], suggesting that the nature of the interaction between *Eriocephalus* and prinia, including the fitness outcomes, may vary geographically.

### 4.1. The plant perspective

Plants whose seeds are dispersed by animals face an evolutionary dilemma: they must attract good seed dispersers while avoiding poor seed dispersers and seed predators. Karoo prinias used large amounts of seed fluff material—material believed to be produced by the plant to enhance seed dispersal by wind [43], and by other bird species [17]—but actively avoided dispersing seeds, suggesting that they are poor seed dispersers. Prinias, however, did disperse some seeds, especially later in the breeding season (electronic supplementary material, figure S4) when seed heads are more easily removed from plants and perhaps are more mature (electronic supplementary material, figure S2). Prinias also dispersed seeds large distances (potentially more than 300 m) and other species of birds likely transport seeds ever farther, particularly relative to dispersal distances by wind [43]. How seeds in prinia nests germinate and grow relative to seeds incorporated into nests of other birds, or not incorporated into bird nests, remains unknown. Dean *et al*. [17] found low germination rates (2.2% of *n* = 724 seeds; which we assume are seed heads that contain multiple seeds, thus per seed germination rates would be lower than 2.2%) of *Eriocephalus* seeds removed from the nests of several species of birds and planted in a nursery setting; however, they did not compare germination rates with seeds that were not incorporated into bird nests. Using Dean *et al*.'s germination rates, the average prinia nest could germinate 0.145 seed heads (6.6 × 0.022 = 0.145), while nests of other species of birds could germinate 1.782 seed heads (81 × 0.022 = 1.782). Assuming that all bird nests provide similar germination success, nests of other bird species could germinate over 12 times as many seeds (equalling, on average, between 1 and 2 plants per nest) compared with nests of prinias.

Seeds incorporated into bird nests may gain several advantages compared with seeds that fall directly to the ground. Dispersal away from parental plants may reduce density dependent seed mortality and improve germination success if seeds are transported to locations that promote growth [44]. Bird nests also have the potential to provide seeds with added nutrients from organic material, especially when nests successfully fledge young, as these nests are often ringed with guano and contain added nitrogen from the feather-sheathing of nestlings [45]. Additionally, seeds incorporated into bird nests may escape from, or have reduced exposure to, terrestrial seed predators (e.g. harvester ants) [17,46], or reduced mortality caused by fungi or bacteria [47,48]. All of these factors have the potential to increase seed germination and early seedling growth, especially in areas of poor soil quality and high risk of seed predation or infection.

Although the Karoo prinia breeding season corresponded well with the production of *Eriocephalus* fluff (figure 2), we do not know if the production of fluff is representative of seed maturity. Over half of the *Eriocephalus* plants that we surveyed had reached the most advanced phenological stage (‘thick fluff’) by 5 October (figure 2), but seed heads on these plants were possibly still developing as their stems were green and well fastened to plants [49]. Similarly, we do not know how seed maturity is linked to germination success. Presumably, seeds removed from plants prior to maturation have reduced germination success and/or survival relative to mature seeds [50]. Establishing the links between fluff production, seed maturity and germination success is an important future step to understand the fitness consequences for *Eriocephalus* plants involved in interactions with birds.

Other birds that harvest *Eriocephalus* seed material for their nests treated seeds differently. On three occasions we witnessed yellow canaries gathering *Eriocephalus* seed material and then crushing seed heads in their bills. In the two yellow canary nests that we examined, 53 of 118 *Eriocephalus* seed heads were broken in half or in thirds, consistent with the seed crushing that we observed in canaries gathering *Eriocephalus* material. By contrast, nests of Cape white-eyes, Cape bulbuls, bokmakierie and chestnut-vented tit-babblers contained many *Eriocephalus* seed heads (mean: 73, range: 0–199), none of which appeared to be damaged. These observations suggest that interactions with some bird species (e.g. yellow canary, Karoo prinia) may be antagonistic, whereas others may be mutualistic [14].

### 4.2. The bird perspective

Life-history theory predicts that investment in each reproductive attempt should decrease as predation risk increases because many attempts are often required to achieve reproductive success [51]. Karoo prinias face extremely high rates of nest predation [24,32], yet they spent a median of 6 days constructing nests and travelled at least as far as 209 m to gather *Eriocephalus* fluff (figure 3). The large amount of time and effort prinias spent gathering *Eriocephalus* fluff suggests that it provides fitness benefits to prinias that build their nests with it.

Below, we outline four hypotheses for potential fitness benefits Karoo prinias, and other bird species, might gain by constructing their nests with *Eriocephalus* material.

#### 4.2.1. Climate

*Eriocephalus* seed material is soft and downy and may help maintain optimal temperature and humidity inside the nest [52,53]. During the early breeding season, and especially at night, ambient temperatures can drop to 8–10°C (electronic supplementary material, figure S1), challenging birds to maintain warm nest temperatures optimal for embryo and nestling development (approx. 36–40°C [52]). Later in the breeding season, and especially in interior valleys away from the coast, temperatures can exceed 35°C, challenging birds to prevent overheating of eggs and nestlings [21]. Breeding sites along the Atlantic coast of South Africa also receive heavy rains during the start of the nesting cycle, when the winter rainy season has not yet ended. Thus, *Eriocephalus* material could help keep nests warm during cool periods and cool during hot periods, maintain stable nest humidity, or help protect eggs, young, and attending females from precipitation.

#### 4.2.2. Predation

*Eriocephalus* material added to bird nests could help reduce the risk of nest predation. Karoo prinias suffer exceptionally high rates of nest predation, with daily nest failure rates of at least 7.5% [24] (V.G.R. 2013, unpublished data). Nest predators at Koeberg are diverse and are thought to use visual and olfactory cues to locate nests. The amount of *Eriocephalus* material, its colour, or its chemical compounds could help reduce nest predation by functioning as a physical barrier that impedes entry of predators to the nest, by concealing nests through crypsis or disruptive patterning [54], by masking olfactory cues associated with nests [55], or by providing an odour that is repulsive to predators [56].

#### 4.2.3. Parasites

Chemical compounds in *Eriocephalus* material could reduce ectoparasite numbers, development and/or diversity on both nestlings or incubating females. *Eriocephalus* compounds have been shown to reduce the growth of microbes and fungi cultured in laboratory settings [57] and to reduce the numbers of nest ectoparasites in species that do not naturally encounter *Eriocephalus* compounds (V.G.R. *et al.* 2012, unpublished data). Karoo prinias continue to add *Eriocephalus* fluff to the interior of their nests during the breeding period, as we would expect if they were trying to maintain levels of aromatic compounds that dissipate over time [58]. Alternatively, the pale colour of *Eriocephalus* material in the nest lining could help parents recognize deleterious foreign objects in the nest, such as ectoparasites or eggs from brood parasites. However, the large amounts of *Eriocephalus* material placed in the base of Karoo prinia nests (electronic supplementary material, figure S3) suggests that fluff does not function to aid in the recognition and exclusion of foreign objects because smaller amounts of *Eriocephalus* lining could achieve the same function.

#### 4.2.4. Social signal

The amount of *Eriocephalus* material brought to the nest by the male or female could signal their quality or condition to their mate [59]. Female Karoo prinias are the primary builders of the nest's grass frame, but both males and females help line the nests. Greater amounts of *Eriocephalus* material brought to the nest could signal a high quality individual, and promote increased investment in breeding by their mates, such as increased clutch size [60], nestling provisioning rates [61] or more vigorous nest defence against predators [59].

All of these hypotheses could improve the survival of nests, the development of eggs and young, and/or reduce physiological, immunological and thermoregulatory demands on attending parents. These hypotheses are not mutually exclusive. *Eriocephalus* might provide birds with multiple benefits, perhaps explaining its use by so many species from diverse avian lineages.

## 5. Conclusion

The interaction between birds and *Eriocephalus* is likely not a simple mutualism because the fitness outcomes for plants appear to depend on the species of birds gathering seed material. Karoo prinias actively avoided seeds when gathering *Eriocephalus* fluff, and dispersed very few seeds relative to the amount of fluff used in their nests. These observations suggest that Karoo prinias are poor dispersers of *Eriocephalus* seeds, especially when compared with other birds that use *Eriocephalus* material in nest construction. Thus, we suspect that *Eriocephalus* plants gain limited dispersal benefits through interactions with prinias. On the other hand, Karoo prinias may benefit from interactions with *Eriocephalus* plants. Despite high rates of nest predation, prinias invest much time and effort in nest construction, frequently travelling outside their territory boundaries to gather *Eriocephalus* fluff. The fitness benefits of incorporating *Eriocephalus* material in nests could be related to nest microclimate, reducing predation or parasitism, or providing a signal of individual condition. While possible fitness benefits to *Eriocephalus* plants and prinias await further study, our observations of this plant–bird interaction suggest that fitness outcomes for *Eriocephalus* plants depend on the species of bird with which plants interact, and that interactions with Karoo prinias result in limited seed dispersal.

## Supplementary Material

Supplementary methods, figures, and statistical summaries

## Data accessibility

Our data are deposited at Dryad: http://dx.doi.org/10.5061/dryad.tv40b [62].
