# Supplementary material for: Fluff-thieving birds sabotage seed dispersal
Source: R Soc Open Sci. 2017 Jan 11;4(1):160538. doi: 10.1098/rsos.160538 (PMC5319318; doi:10.1098/rsos.160538)
Supplement: Supplementary methods, figures, and statistical summaries [file rsos160538supp1.docx]

**Fluff-thieving birds sabotage seed dispersal**

Supplemental information: methods, figures, references, description of the phenological categories of *Eriocephalus* plants, and detailed statistical summaries of hurdle and GLM models

**Detailed methods for quantifying *Eriocephalus* fluff on the interior and exterior of nests**

***Nest interiors.*** We estimated the proportion of the nest interior that was covered in *Eriocephalus* fluff using ImageJ software [1]. We calculated proportions by dividing the total area of pale colored material (primarily *Eriocephalus* fluff) on the nest interior by the total area of the nest interior. We estimated the area of *Eriocephalus* fluff and nest interior for each nest by photographing cut-in-half nests against a dark background; we took all photos using a Panasonic Lumix FZ150 camera (Panasonic Corporation, Japan) and included a 10cm ruler beside all nests for scale. We opened photos in ImageJ and: (i) converted color images to 8-bit grey scale, (ii) set the scale for each image, (iii) adjusted the pixel threshold to capture the total nest area, and then the area of pale colored nest lining, (iv) converted the adjusted image to binary so that the nest or the pale lining became dark colored, (v) summed the area of dark color representing the nest or pale lining material; this procedure follows Reinking [2]. While we took all nest pictures against a dark background with similar ambient light levels, the pixel threshold used to quantify *Eriocephalus* material inside each nest was slightly different because of small differences in exposure levels, ambient light levels, and materials used in nest construction. To control for any differences in measurement techniques among observers, VGR made all measures in ImageJ; thus, any biases in measurement should be similar across all nests. VGR scored a subset of 15 of the 117 nests five times, each on different days, to assess the repeatability of threshold measures; repeatability was high (Intraclass correlation coefficient: 0.93, 95% CI range 0.861–0.972, *F*_14,60_ = 67.1, *p* < 0.00001).

***Nest exteriors.*** We estimated the proportion of the nest exterior that was covered by *Eriocephalus* fluff using nests that were cut in half. We placed nest halves with the nest exterior facing up, then sectioned each nest into thirds where the bottom third represented the base of the nest, the middle third the nest-walls, and the top third the nest-roof. We visually estimated the proportion of *Eriocephalus* fluff in each nest section, covering the other sections with gray-colored cardboard. All estimates were made by VGR. We then averaged the estimated proportions of *Eriocephalus* material visible on the exterior base, walls and roof of each nest to generate our measure of the proportion of *Eriocephalus* material visible on the nest exterior.

**Statistical analyses of *Eriocephalus* seeds and fluff in Karoo prinia nests**

For the analysis of *Eriocephalus* seeds in prinia nests, we excluded nests that contained no *Eriocephalus* material (and thus no seeds). We examined the factors that influenced the number of *Eriocephalus* seeds in prinia nests using hurdle models that are suitable for zero-inflated count data, following Zuur et al. [3], chapter 11. Hurdle models separately test the effects of predictor variables on the zero versus non-zero (bivariate) component of the response variable, and on variation in the non-zero component of the response variable [3]. In our analysis, the response variable was the number of *Eriocephalus* seeds in nests and our predictor variables were: proximity of the nest to the nearest *Eriocephalus* bush, first egg date, minimum ambient temperature, nest bush species, nest height, and the number of days the nest remained active (see main text for rationale for including these variables in the model). Several variables had distributions that were significantly different from a normal distribution (following Shapiro-Wilk tests). We transformed these variables prior to analysis to increase the likelihood that our data fit the assumptions of our model: proximity to closest *Eriocephalus* bush [natural logarithm (closest *Eriocephalus* bush + 3)], first egg date (first egg date^0.2), nest height (nest height^0.1), number of days a nest remained active (-1/(days active ^0.3)), and minimum ambient temperature (temperature^0.7). For nest bush species, we grouped all bush species with fewer than 4 nests into a single category “other”, leaving a total of eight categories for bush species.

For analyses of *Eriocephalus* fluff, we ran four models each using a different measure of *Eriocephalus* fluff as our dependent variable [i.e., total mass of *Eriocephalus* fluff, average depth of fluff in the base of nests, the proportion of pale colored material (primarily *Eriocephalus* fluff) on the interior of the nest, and the proportion of fluff on the exterior of nests]. We examined these four different measures of *Eriocephalus* fluff separately because *Eriocephalus* material in different locations of the nest may have different functions, and because birds may prioritize the placement of *Eriocephalus* material in nests depending on the availability of material. For analyses of depth and mass of *Eriocephalus* fluff, we used hurdle models, and for analyses of the amount of *Eriocephalus* material on the interior and exterior of nests, we used Generalized Linear Models (GLMs).

Both *Eriocephalus* depth and mass were bimodal, with peaks at zero and near the mid-point of each variable. We converted these continuous variables with decimal measures into whole-number integers (similar to count data), so that we could run hurdle models. For depth of *Eriocephalus* fluff, we binned measures into 2mm intervals as follows: nests with < 2mm of *Eriocephalus* fluff were scored as 0, 2-4mm as 1, 4-6mm as 2, etc.; this binning scale resulted in bins from 0–13, spanning *Eriocephalus* depth measures 0–27mm. For total mass of *Eriocephalus* fluff, we binned measures into 0.25g intervals with nests having <0.25g scored as 0, 0.26-0.5g as 1, 0.51-0.75g as 2, 0.76-1.0g as 3, etc.; this binning scale resulted in bins ranging from 0–13, spanning *Eriocephalus* mass measures 0–3.4g. Binning continuous variables risks creating artificial categories; thus, we ran both of these models with different binning categories (i.e., for *Eriocephalus* depth, we used 1mm and 3mm bins; and for *Eriocephalus* mass we used 0.1g and 0.5g bins); bin size did not change the best performing models or which predictor variables were important.

We excluded one nest from analyses of the depth of *Eriocephalus* fluff because the depth of fluff was over 5 standard deviations larger than the mean of all other nests (mean ± 1sd of nests with outlier excluded 12.9 ± 7.7mm, depth of fluff of outlier 52.5mm); no other prinia nests approached this value (max depth of *Eriocephalus* fluff in all other nests 27mm).

For GLMs, we transformed the amount of *Eriocephalus* material on the exterior and interior of nests prior to analyses because both variables had right skewed distributions and were significantly different from a normal distribution (following Shapiro-Wilk tests). We used (exterior^0.6) to normalize the exterior measures of the nest. Common transformations, however, could not normalize the interior measures, so we used the *Continuous Fit > All* function in JMP [4] that ranks different transformations (using AIC) in terms of their ability to normalize data. For nest interior measures, we chose the best-performing transformation (lowest AIC): a Johnson Su transformation with the following formula:

$$ArcSinH\left( \frac{(interior-0.967)}{0.0232} \right)*1.666+5.190$$

For all four analyses of *Eriocephalus* fluff, we used a single measure of fluff as the dependent variable (i.e., depth of fluff in nest base, total mass, proportion pale material in the interior, proportion fluff on the exterior) and, in all analyses, we used the same predictor variables: proximity to the nearest *Eriocephalus* bush, first egg date, minimum ambient temperature, nest bush species, nest height, and the number of days the nest remained active. As before, we transformed predictor variables prior to analyses to better fit the assumptions of our models using the following transformations: first egg date [natural logarithm (first egg date)], distance to closest *Eriocephalus* bush (closest bush^0.3), minimum ambient temperature (temperature^0.7), number of days a nest remained active [-1/(days active^0.3)], nest height (height^0.2). For nest bush species, we again grouped all bush species with fewer than 4 nests into a single category “other”.

We identified the best-performing models using AIC, corrected for small sample sizes (AICc), and the *dredge* command in package MuMIn [5] in R [6]. Prior to model selection, we checked the assumptions of hurdle models and GLMs following Zuur et al. [3]. For hurdle models, we plotted Pearson’s residuals against predictor variables to ensure that model residuals showed no patterns with predictor variables. For GLMs, we plotted model residuals against predictor variables and checked that the distribution of model residuals did not deviate from normality using Shapiro-Wilk tests. For both hurdle models and GLMs, we checked for similar residual variance among the categorical predictor variable “nest bush species” using Bartlett’s tests. We present best-performing models (those with ΔAICc<2) and averaged parameter estimates, which take into account the frequency that predictor variables were included in best-performing models and the fit of those models. We report standardized effect sizes for all analyses using the formula: [(1 sd of the predictor variable)/(1 sd of the response variable)*the slope of predictor variable]. This procedure follows [7] and incorporates the strength of the relationship between a predictor variable and the response variable using standardized units (sd) across variables. Finally, because our different measures of *Eriocephalus* fluff were not independent, we corrected for false discovery rates following Benjamini et al. [8] and Pike [9].

**Supplementary figures**

**Figure S1.** Environmental conditions at the Koeberg Nature Reserve. Grey band spans the range of average maximum and minimum temperatures (from years 2002–2012), and black line shows average ± 1SE rainfall (from years 1980–2013) throughout the year. Black box and dotted line in lower right of plot shows breeding activity of Karoo prinias, with most breeding activity indicated by the black box and less frequent breeding activity indicated by the dotted line [10].


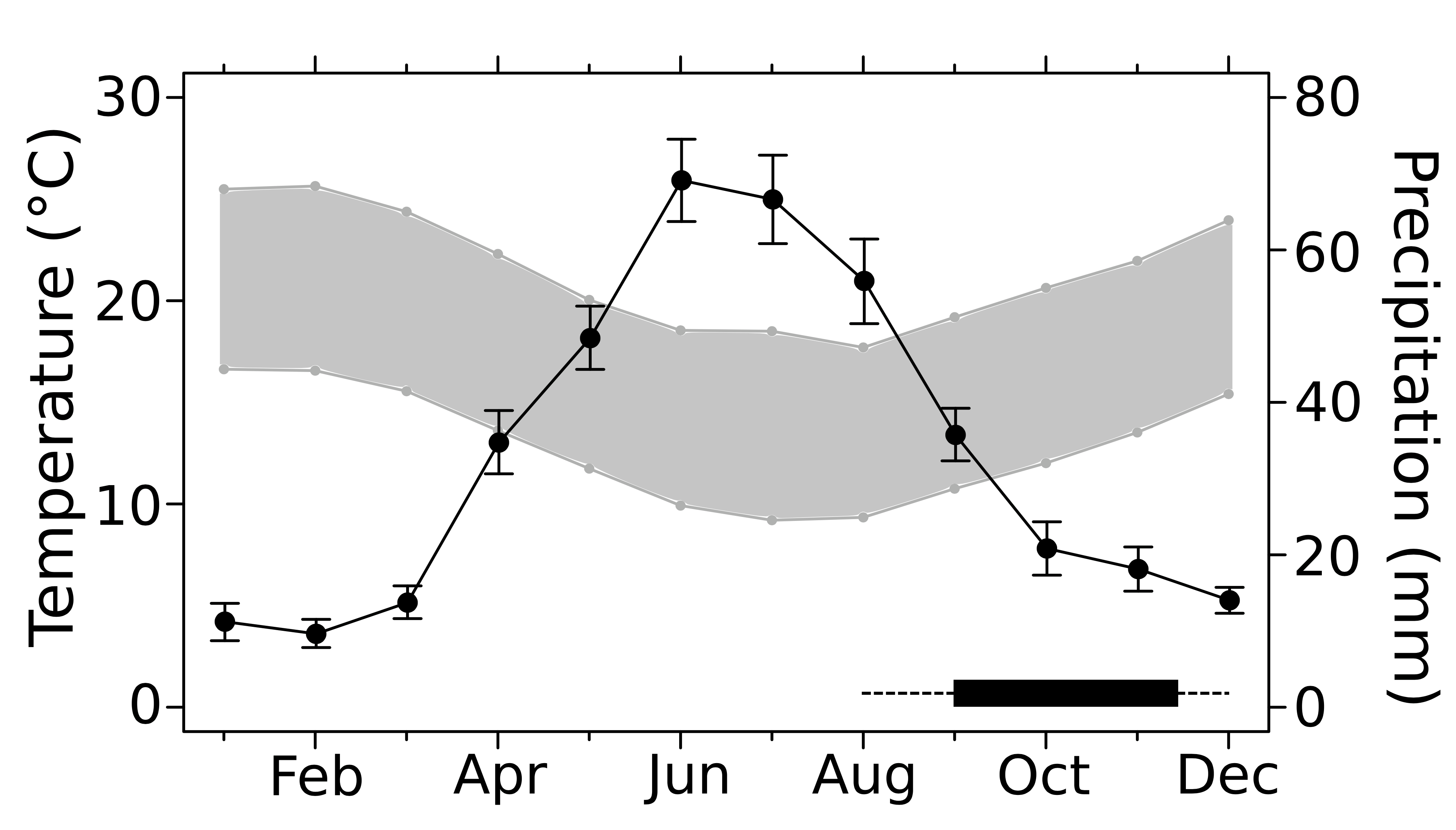


**Figure S2.** Number of seed heads removed from *Eriocephalus* plants over the season. Box plots summarize the number of seeds heads removed after plucking at the fluff of 100 *Eriocephalus* seed heads per plant for 10 plants during the early (26 Aug), middle (1 Oct), and late (3 Nov) stages of the Karoo prinia breeding season. Each box represents data from 100 plucks from 10 plants, for a total of 1000 plucks for each date. We returned to the same plants for each plucking event. The number of seed heads removed with plucking increased with date, and all plucking events differed from each other in the number of seed heads removed (glmm; *p*<0.001 for all intercepts). Pictures illustrate plucking events that did (top) and did not (bottom) result in the removal of seed heads.

**Figure S3.** Measures of wall thickness for Karoo prinia nests. Nest-wall thickness for 10 measures evenly distributed around the perimeter of Karoo prinia nests that were cut in half (*n* = 122). Measures include the grass frame and nest lining, which was primarily *Eriocephalus* fluff. Photo illustrates the location of the 10 measures and indicates the nest entrance, roof, and bottom of nest.

**Figure S4.** Factors influencing the number of *Eriocephalus* seed heads in Karoo prinia nests. (A) Prinia nests that are closer to *Eriocephalus* plants and (B) nests constructed later in the breeding season contained more *Eriocephalus* seed heads. Number of nests examined for each plot (*n* = 99).

**References**

1. Rasband WS. 1997–2014 *ImageJ.* U. S. National Institutes of Health, Bethesda, Maryland, USA.

2. Reinking L. 2007 *Examples of image analysis using ImageJ.* <http://rsb.info.nih.gov/ij/docs/pdfs/examples.pdf> (accessed September 2014).

3. Zuur AF, Ieno EN, Walker NJ, Saveliev AA, Smith GM. 2009 *Mixed effects models and extensions in ecology with R.* New York, NY: Springer.

4. JMP. 2007 Version 11.0, SAS Institute Inc., Cary, NC.

5. Bartoń K. 2014 *MuMIn: Multi-model inference* (R package version 1.10.0). http://CRAN.R-project.org/package=MuMIn

6. R Core Team. 2014 *R: A language and environment for statistical computing* Vienna, Austria: R Foundation for Statistical Computing.

7. Nieminen P, Lehtiniemi H, Vähäkangas K, Huusko A, Rautio A. 2013 Standardized regression coefficient as an effect size index in summarizing findings in epidemiological studies. *Epidemiol Biostat Public Health.* **10,** e8854-1– e8854-15.

8. Benjamini Y, Krieger A, Yekutieli D. 2006 Adaptive linear step-up procedures that control the false discovery rate. *Biometrika*. **93**, 491–507.

9. Pike N. 2011 Using false discovery rates of multiple comparisons in ecology and evolution. *Method Ecol Evol*. **2**, 278–282.

10. Tarboton W. 2011 *Roberts nests and eggs of southern African birds.* Cape Town, South Africa: John Voelcker Bird Book Fund.

ESM movie caption.

Video of an adult Karoo prinia gathering fluff from a *Eriocephalus racemosus* plant at the Koeberg Nature Reserve, Western Cape Province, South Africa. *Eriocephalus* plants have flexible branches that bend easily in the wind, making the acquisition of fluff challenging for prinias. This prinia gathers a bill full of fluff from seed heads held in place with its toes, a behavior that likely reduces the number of seed heads removed from *E. racemosus* plants.

**Table S1**. Descriptions of the phenological categories of *Eriocephalus*.

| Stage | Description |
| --- | --- |
| 1 | Bud. <50% of the buds are blooming. |
| 2 | Early flowering. >50% of the buds are blooming, flowers just beginning to open giving bush a slightly rosy appearance. |
| 3 | Peak flowering. Nearly all buds are blooming. Overall bush has rosy and yellowish tints from blossoms. Fluff just beginning to develop but its length does not exceed the length of flowering heads. |
| 4 | Late flowering. Flowers still visible, but the fluff is beginning to develop. Fluff appears as sparse plumes, which are generally shorter than flowering heads. |
| 5 | Sparse fluff. Flower heads still visible but surrounded by sparse plumes of fluff. Length of fluff generally equals length of flower heads. |
| 6 | Early fluff. Individual flower heads are recognizable but nearly invisible because of thick, short fluff surrounding them. Bush begins to appear white. |
| 7 | Medium fluff. Center of flower head has brown dimple and length of fluff exceeds length of flower head. Seed heads generally appear white, but fluff is often projected toward the tip of the flower head. Bush has overall white appearance. |
| 8 | Thick fluff. Bush has blazing white appearance. Fluff is so thick that it obscures leaves and often makes recognition of individual flower heads difficult. Flower heads continue to have brown dimple in their center. Fluff appears fully developed and often extends away from the seed head in a globe like fashion. |

**Table S2.** Significant predictor variables influencing the number of *Eriocephalus* seed heads and amount of *Eriocephalus* fluff in Karoo prinia nests. Summaries of analyses for: *Eriocephalus* seeds in nests, mass of *Eriocephalus* fluff, average depth of *Eriocephalus* fluff in the base of nests, and the proportion of *Eriocephalus* fluff in the interior and exterior of nests. Analyses of seeds, mass, and depth used hurdle models that examine the variation between nests constructed with and without *Eriocephalus* material (ne), and the variation in nests with *Eriocephalus* material (we), separately. Analyses of the proportion of *Eriocephalus* fluff in the interior and exterior of Karoo prinia nests used GLMs. Table shows p-values and slopes (slopes given in parentheses) of predictor variables significantly correlated with *Eriocephalus* material in nests. No analyses recognized nest height, bush species, or ambient temperature (after controlling for first egg date) as important predictors of the amount of *Eriocephalus* seeds or fluff in nests. Numbers of nests included in each analysis are presented in each column (range 99–105).

|  | Seeds  *n* = 99 | Fluff mass  *n* = 105 | Average depth  *n* = 104 | Interior  *n* = 105 | Exterior  *n* = 105 |
| --- | --- | --- | --- | --- | --- |
| Proximity of nest to bush | 0.0009  (ne -1.00) | 0.0023  (we -0.16) 0.0160  (ne -5.39) | 0.0001  (we -0.18) 0.0020  (ne -4.41) | 0.0001  (-0.40) | 0.0001  (-1.30) |
| First egg date | 0.0010  (we 9.55) | 0.0001  (we -4.26) | 0.0170  (we -1.9) |  |  |
| Number of days active |  |  |  | 0.0001  (14.40) |  |

Table S3A. Best-performing models (ΔAICc < 2) for the number of *Eriocephalus* seeds in Karoo prinia nests as a function of: first egg date, proximity of nests to nearest *Eriocephalus* bush, ambient temperature, number of days nests remained active, nest height and bush species in which nests were placed. We used hurdle models that examine the variation between nests with and without *Eriocephalus* seeds (ne), and the variation among nests with *Eriocephalus* seeds (we), separately. Sample sizes: nests without seeds *n* = 19; nest with seeds: *n* = 80; total nests: *n* = 99.

| Model | df | AICc | ΔAICc | Weight | Log likelihood |
| --- | --- | --- | --- | --- | --- |
| First egg date (we) + proximity (ne) | 5 | 573.09 | 0 | 0.17 | -281.22 |
| First egg date (we) + proximity (ne) + proximity (we) | 6 | 573.16 | 0.07 | 0.16 | -280.12 |
| First egg date (we) + proximity (ne) + ambient temperature (ne) | 6 | 573.97 | 0.88 | 0.11 | -280.53 |
| First egg date (we) + proximity (ne) + proximity (we) + ambient temperature (ne) | 7 | 574.08 | 0.99 | 0.10 | -279.43 |
| First egg date (we) + proximity (ne) + first egg date (ne) | 6 | 574.30 | 1.21 | 0.09 | -280.69 |
| First egg date (we) + proximity (ne) + proximity (we) + first egg date (ne) | 7 | 574.41 | 1.32 | 0.09 | -279.59 |
| First egg date (we) + proximity (ne) + ambient temperature (we) | 6 | 574.47 | 1.38 | 0.08 | -280.78 |
| First egg date (we) + proximity (ne) + days active (we) | 6 | 574.76 | 1.67 | 0.07 | -280.92 |
| First egg date (we) + proximity (ne) + proximity (we) + days active (we) | 7 | 574.81 | 1.72 | 0.07 | -279.79 |
| First egg date (we) + proximity (ne) + proximity (we) + ambient temperature (we) | 7 | 574.96 | 1.87 | 0.06 | -279.87 |

Table S3B. Parameter estimates averaged from all best-performing models (ΔAICc < 2) in Table S2A, for factors influencing the number of *Eriocephalus* seeds in Karoo prinia nests. For this and all subsequent tables ES is a measure of effect size calculated as: ((sd in predictor variable)/(sd in response variable))*slope (Estimate) of the predictor variable, following [7]. SE is the standard error of the Estimate (e.g., slope) of each predictor variable.

| Parameter | Estimate | ES | SE | *z* | *p* |
| --- | --- | --- | --- | --- | --- |
| First egg date (we) | 9.55 | 0.039 | 2.91 | 3.29 | **0.0010** |
| Proximity (ne) | -1.00 | -0.139 | 0.30 | 3.30 | **0.0009** |
| Proximity (we) | 0.08 | 0.011 | 0.11 | 0.70 | 0.48 |
| Ambient temperature (ne) | 0.07 | 0.008 | 0.19 | 0.37 | 0.71 |
| First egg date (ne) | 1.32 | 0.005 | 4.22 | 0.31 | 0.75 |
| Ambient temperature (we) | 0.02 | 0.002 | 0.08 | 0.26 | 0.80 |
| Days active (we) | -0.07 | -0.001 | 0.29 | 0.24 | 0.81 |

Table S4A. Best-performing models (ΔAICc < 2) for the average depth of *Eriocephalus* fluff in the base of Karoo prinia nests as a function of: first egg date, proximity of nests to nearest *Eriocephalus* bush, ambient temperature, number of days nests remained active, nest height and bush species in which nests were placed. We used hurdle models that examine the variation between nests with and without *Eriocephalus* fluff (ne), and the variation among nests with *Eriocephalus* fluff (we), separately. Sample sizes: nests without fluff *n* = 16; nest with fluff *n* = 88; total number of nests: *n* = 104.

| Model | df | AICc | ΔAICc | Weight | Log likelihood |
| --- | --- | --- | --- | --- | --- |
| Proximity (we) + first egg date (we) + proximity (ne) | 5 | 445.23 | 0 | 0.52 | -217.31 |
| Proximity (we) + first egg date (we) + proximity (ne) + days active (we) | 6 | 446.62 | 1.39 | 0.26 | -216.88 |
| Proximity (we) + first egg date (we) + proximity (ne) + nest height (ne) | 6 | 446.96 | 1.73 | 0.22 | -217.04 |

Table S4B. Parameter estimates averaged from all best-performing models (ΔAICc < 2) in Table S3A, for factors influencing the average depth of *Eriocephalus* fluff in the base of Karoo prinia nests. *P*-adj corrects for false discovery rates following Benjamini et al. [8], because we measured four different, but related, components of *Eriocephalus* fluff in prinia nests.

| Parameter | Estimate | ES | SE | *z* | *p* | *p*-adj |
| --- | --- | --- | --- | --- | --- | --- |
| Proximity (we) | -0.18 | -0.059 | 0.04 | 4.33 | **<0.0001** | **<0.0001** |
| First egg date (we) | -1.90 | -0.031 | 0.70 | 2.71 | **0.0068** | **0.017** |
| Proximity (ne) | -4.41 | -1.476 | 1.27 | 3.46 | **0.0005** | **0.002** |
| Days active (we) | -0.41 | -0.003 | 1.10 | 0.37 | 0.71 | 0.62 |
| Nest height (ne) | 0.80 | 0.027 | 2.86 | 0.28 | 0.78 | 0.62 |

Table S5A. Best-performing models (ΔAICc < 2) for the total mass of *Eriocephalus* fluff in Karoo prinia nests as a function of: first egg date, proximity of nests to nearest *Eriocephalus* bush, ambient temperature, number of days nests remained active, nest height and bush species in which nests were placed. We used hurdle models that examine the variation between nests with and without *Eriocephalus* fluff (ne), and the variation among nests with *Eriocephalus* fluff (we), separately. Sample sizes: nests without fluff *n* = 14, nest with fluff *n* = 91; Total number of nests: *n* = 105.

| Model | df | AICc | ΔAICc | Weight | Log likelihood |
| --- | --- | --- | --- | --- | --- |
| First egg date (we) + proximity (we) + proximity (ne) + days active (we) | 6 | 395.32 | 0 | 0.13 | -191.23 |
| First egg date (we) + proximity (we) + proximity (ne) + days active (we) + nest height (we) | 7 | 395.56 | 0.23 | 0.12 | -190.20 |
| First egg date (we) + proximity (we) + proximity (ne) | 5 | 395.87 | 0.55 | 0.10 | -192.63 |
| First egg date (we) + proximity (we) + proximity (ne) + days active (we) + days active (ne) | 7 | 396.66 | 1.33 | 0.07 | -190.75 |
| First egg date (we) + proximity (we) + proximity (ne) + days active (we) + nest height (ne) | 7 | 396.73 | 1.40 | 0.07 | -190.79 |
| First egg date (we) + proximity (we) + proximity (ne) + nest height (we) | 6 | 396.88 | 1.56 | 0.06 | -192.01 |
| First egg date (we) + proximity (we) + proximity (ne) + days active (we) + days active (ne) + nest height (we) | 8 | 396.94 | 1.62 | 0.06 | -189.72 |
| First egg date (we) + proximity (we) + proximity (ne) + days active (we) + first egg date (ne) | 7 | 396.99 | 1.67 | 0.06 | -190.92 |
| First egg date (we) + proximity (we) + proximity (ne) + days active (we) + nest height (we) + nest height (ne) | 8 | 397.01 | 1.69 | 0.06 | -189.75 |
| First egg date (we) + proximity (we) + proximity (ne) + ambient temperature (we) | 6 | 397.08 | 1.76 | 0.06 | -192.11 |
| First egg date (we) + proximity (we) + proximity (ne) + days active (ne) | 6 | 397.15 | 1.83 | 0.05 | -192.15 |
| First egg date (we) + proximity (we) + proximity (ne) + days active (we) + ambient temperature (we) | 7 | 397.16 | 1.84 | 0.05 | -191.00 |
| First egg date (we) + proximity (we) + proximity (ne) + nest height (ne) | 6 | 397.22 | 1.90 | 0.05 | -192.18 |
| First egg date (we) + proximity (we) + proximity (ne) + days active (we) + nest height (we) + first egg date (ne) | 8 | 397.27 | 1.95 | 0.05 | -189.89 |

Table S5B. Parameter estimates averaged from all best-performing models (ΔAICc < 2) in Table S4A, for factors influencing the total mass of *Eriocephalus* fluff in Karoo prinia nests. *P*-adj corrects for false discovery rates following Benjamini et al. [8], because we measured four different, but related, components of *Eriocephalus* fluff in prinia nests. Sample sizes: *n* = 105 nests.

| Parameter | Estimate | ES | SE | *z* | *p* | *p*-adj |
| --- | --- | --- | --- | --- | --- | --- |
| First egg date (we) | -4.26 | -0.084 | 0.92 | 4.62 | **<0.0001** | **<0.0001** |
| Proximity (we) | -0.16 | -0.065 | 0.05 | 3.38 | **0.0007** | **0.0023** |
| Proximity (ne) | -5.39 | -2.176 | 1.90 | 2.84 | **0.0046** | **0.016** |
| Days active (we) | 2.23 | 0.018 | 2.20 | 1.01 | 0.31 | 0.57 |
| Nest height (we) | 0.17 | 0.007 | 0.32 | 0.55 | 0.59 | 0.62 |
| Days active (ne) | 9.37 | 0.074 | 31.30 | 0.30 | 0.76 | 0.62 |
| Nest height (ne) | 0.97 | 0.226 | 3.31 | 0.29 | 0.77 | 0.62 |
| First egg date (ne) | 1.00 | 0.019 | 4.87 | 0.21 | 0.84 | 0.62 |
| Ambient temperature (we) | -0.01 | -0.002 | 0.03 | 0.22 | 0.83 | 0.62 |

Table S6A. Best-performing models (ΔAICc < 2) for the proportion of *Eriocephalus* fluff on the nest exterior and pale colored material (primarily *Eriocephalus* fluff) on the nest interior of Karoo prinia nests as a function of: first egg date, proximity of nests to the nearest *Eriocephalus* bush, ambient temperature, number of days a nest remained active, nest height, and bush species in which nests were placed. Both models used GLMs and both have samples sizes: *n* = 105.

| Model Exterior | df | AICc | ΔAICc | Weight | Log likelihood |
| --- | --- | --- | --- | --- | --- |
| Proximity + days active | 4 | 451.92 | 0 | 0.38 | -221.76 |
| Proximity + days active + first egg date | 5 | 452.51 | 0.59 | 0.28 | -220.95 |
| Proximity + first egg date | 4 | 453.52 | 1.61 | 0.17 | -222.56 |
| Proximity + days active + nest height | 5 | 453.64 | 1.73 | 0.16 | -221.52 |
| Model Interior |  |  |  |  |  |
| Proximity + days active + first egg date | 5 | 248.25 | 0 | 0.55 | -118.82 |
| Proximity + days active + ambient temperature | 5 | 249.99 | 1.73 | 0.23 | -119.69 |
| Proximity + days active + first egg date + nest height | 6 | 250.04 | 1.79 | 0.22 | -118.59 |

Table S6B. Parameter estimates averaged from all best-performing models (ΔAICc < 2) in Table S5A, for factors influencing the proportion of *Eriocephalus* fluff on the exterior and interior of Karoo prinia nests. *P*-adj corrects for false discovery rates following Benjamini et al. [8], because we measured four different, but related, components of *Eriocephalus* fluff in prinia nests. Sample sizes: *n* = 105 nests.

| Parameters Exterior | Estimate | ES | SE | *z* | *p* | *p*-adj |
| --- | --- | --- | --- | --- | --- | --- |
| Proximity | -1.30 | -0.617 | 0.16 | 7.92 | **<0.0001** | **<0.0001** |
| Days active | 13.91 | 0.156 | 9.90 | 1.40 | 0.16 | 0.36 |
| First egg date | -2.16 | -0.050 | 3.29 | 0.65 | 0.51 | 0.62 |
| Nest height | 0.17 | 0.008 | 0.74 | 0.23 | 0.82 | 0.62 |
| Parameters Interior |  |  |  |  |  |  |
| Proximity | -0.40 | -0.502 | 0.06 | 6.46 | **<0.0001** | **<0.0001** |
| Days active | 14.40 | 0.356 | 3.20 | 4.44 | **<0.0001** | **<0.0001** |
| First egg date | 2.07 | 0.166 | 1.59 | 1.30 | 0.19 | 0.39 |
| Ambient temperature | 0.03 | 0.031 | 0.07 | 0.45 | 0.65 | 0.62 |
| Nest height | 0.09 | 0.011 | 0.33 | 0.27 | 0.79 | 0.62 |
